# A Novel GH Deficient Rat Model Reveals Cross‐Species Insights Into Aging

**DOI:** 10.1111/acel.70126

**Published:** 2025-06-05

**Authors:** Soe Maung Maung Phone Myint, Alexander Tate Lasher, Kaimao Liu, Aron M. Geurts, Steven N. Austad, Liou Y. Sun

**Affiliations:** ^1^ Department of Biology University of Alabama at Birmingham Birmingham Alabama USA; ^2^ Department of Physiology Medical College of Wisconsin Milwaukee Wisconsin USA

**Keywords:** aging, endocrinology, gender differences, insulin/IGF‐1 signaling, lifespan, longevity, metabolic rate, rat models

## Abstract

Multiple studies in mice with genetically disrupted growth hormone (GH) signaling have demonstrated that such disruption results in reduced body size, robustly increased longevity (> 50% in some cases), and improvements across multiple health parameters. However, it remains unclear how generalizable these findings are across mammals. Evidence in rats is limited and inconsistent. These conflicting results highlight the need for further investigation into the role of GH signaling in longevity across species. To address this gap, we developed a novel GH‐deficient rat model using CRISPR/Cas9 technology to introduce a 10 bp deletion in exon 3 of the gene encoding rat GH‐releasing hormone (GHRH) yielding a non‐functional GHRH product. Physiological characterization of GHRH knockout (KO) rats revealed that they were half the body weight of wild‐type controls. Additionally, relative to controls, they displayed an increased percent body fat, enhanced insulin sensitivity, reduced circulating insulin‐like growth factor I (IGF‐I) concentration, and a decreased reliance on glucose oxidation for energy metabolism, as determined by indirect calorimetry. Analysis of the gut microbial community in adult GHRH‐KO rats further revealed a less diverse male microbiome, but a more diverse female KO microbiome compared to controls. Collectively, these findings demonstrate that multiple aspects of the GH activity‐deficient phenotype, well‐documented in mice, are faithfully recapitulated in our rat model. Therefore, the GHRH‐deficient rat model represents a valuable new tool for advancing our understanding of the role of GH signaling in aging processes.

## Graphical abstract

Mouse studies show disrupted GH signaling reduces body size, increases longevity, and improves health. We developed a CRISPR/Cas9 GH‐deficient rat model to address inconsistent rat evidence. GH‐deficient rats mirrored these findings, showing reduced body weight, increased body fat, enhanced insulin sensitivity, and altered gut microbiomes.

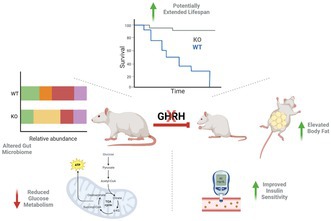

## 1 Introduction

As developed nations face aging populations, a greater portion of life is expected to be spent suffering from chronic age‐related diseases. Up to 20% of life is affected by these conditions, with the burden rising faster in women, making aging a significant public health concern (Garmany and Terzic 2024; Partridge et al. 2018). A key avenue for extending lifespan in mammals involves genetic disruption of growth hormone (GH) activity. GH‐deficient Ames and Snell dwarf mice, as well as GH‐releasing hormone (GHRH) receptor‐deficient “Little” mice, exhibit extended lifespans compared to controls (Brown‐Borg et al. 1996; Flurkey et al. 2002, 2001). Similar results are observed in mice with targeted deletions of GHRH, GH receptors, or GH genes (Adkins‐Jablonsky et al. 2024; Coschigano et al. 2003; Sun et al. 2013). The reproducibility of these findings across various genetic backgrounds underscores the GH‐deficient system as a valuable model for studying mechanisms of healthy aging.

Beyond longevity, GH‐deficient mice experience improved health, including lower mortality rates, enhanced coordination, and better cognitive function (Arum et al. 2013; Kinney et al. 2001; Koopman et al. 2016; Sun et al. 2017). These mice exhibit unique physiological traits such as reduced IGF‐I levels, increased adiposity, enhanced insulin sensitivity, and decreased reliance on glucose oxidation (Icyuz et al. 2020; Westbrook et al. 2009; Wiesenborn et al. 2014; Zhang et al. 2020). However, GH deficiency's effects on lifespan in other mammals are inconsistent. While hypophysectomized and spontaneous dwarf rats show lifespan extensions (Everitt et al. 1980; Sasaki et al. 2013), Lewis dwarf rats display no change (Sonntag et al. 2005), and anti‐sense GH transgene expressing rats show either reduced or extended lifespans depending on the level of transgene expression (Shimokawa et al. 2002). These discrepancies highlight the need to investigate GH signaling in species beyond mice.

Rats offer key advantages over mice for aging research, including more human‐like end‐of‐life pathology, complex social behaviors, suitability for cognitive testing, and better glucose metabolism assessments (Carter et al. 2020; Yang et al. 2004). However, existing rat models rely on surgical intervention, spontaneous mutations, or anti‐sense transgene expression, making it difficult to isolate GH's role in aging. To address this, we utilized CRISPR/Cas9 to create a GHRH knockout rat model. Our in‐depth physiological analysis confirms that these rats replicate key GH‐deficient traits observed in mice, offering a novel platform for studying GH's impact on aging.

## 2 Results

**FIGURE 1:**
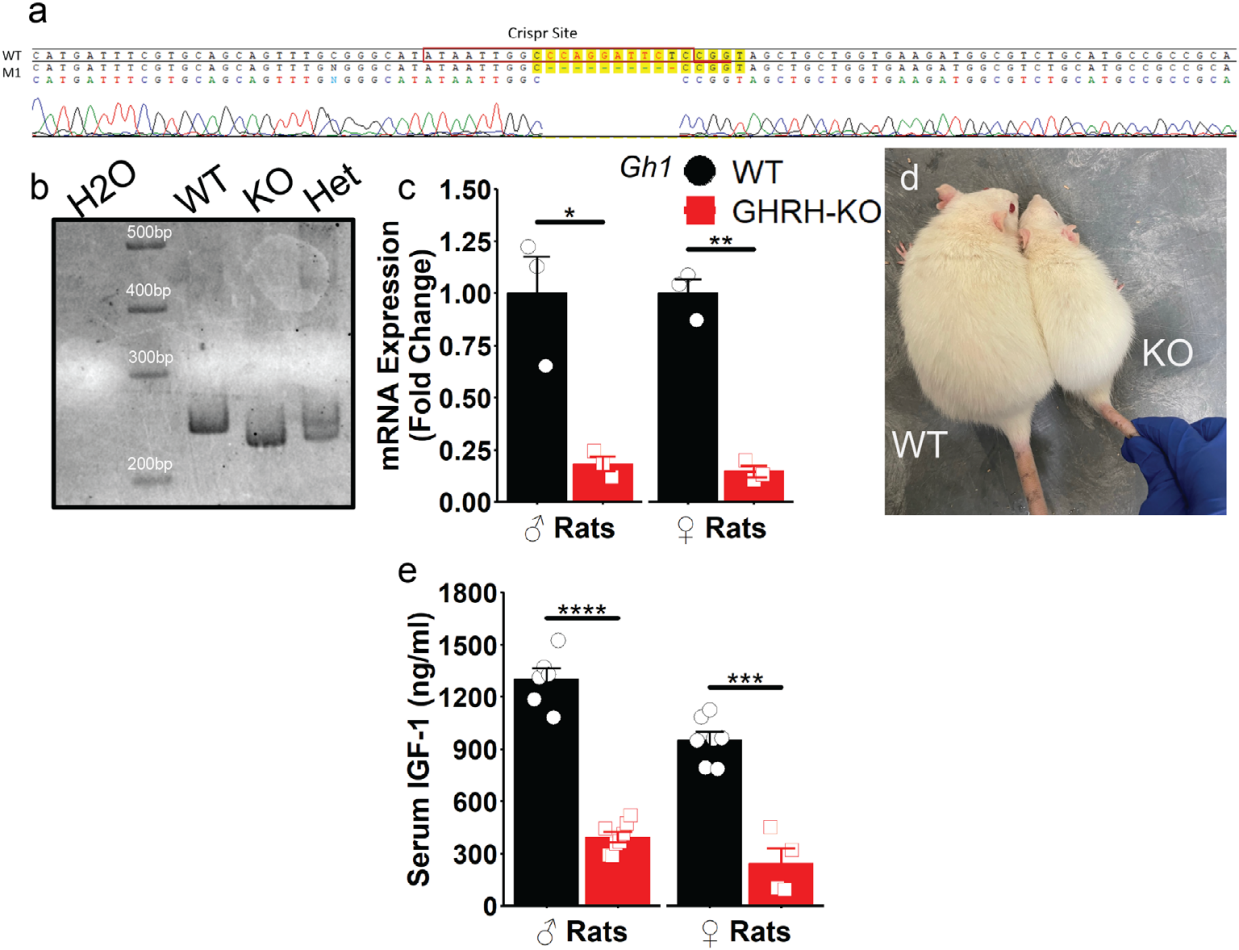
Development of a GHRH knockout rat model. DNA sequence chromatogram of the WT and mutant GHRH gene (a). Genotyping of tail DNA by PCR using primers spanning exon 3 of the gene coding for rat GHRH reveals a 10 bp deletion in mutant rats (b). mRNA abundance of *Gh1* transcripts in the pituitary of 3–4 month‐old rats, with *Actb* used as an endogenous control. Representative image of adult female rats (d). Serum IGF‐1 levels, assessed by ELISA in 1‐year‐old male and female rats as indicated (e). Data presented as mean ± SEM with points representing individual rats. *p < 0.05, **p < 0.01, ***p < 0.001, ****p < 0.0001 as determined by two‐tailed *n*‐test (c, e). *N* = 3–8 per group.

Utilizing CRISPR/Cas9‐mediated gene editing, we generated rats carrying a 10 bp deletion in exon 3 of the rat *Ghrh* gene (Figure S1 and Figure 1a,b). Predicted translation of the resulting sequence yields MPLWVFFVLLTLTSGSHCSLPPSPPFRVRRHADAIFTSSYRANYMPANCCTKS, a loss of 51 amino acids. Rats that were homozygous for this mutation, termed GHRH‐KO rats, displayed significantly reduced pituitary *Gh1* mRNA abundance (Figure 1c), suggesting successful GH interruption. Adult body size was notably reduced in GHRH‐KO rats (Figure 1d), and assessment of circulating IGF‐I levels was significantly lower in GHRH‐KO rats (Figure 1e).

**FIGURE 2:**
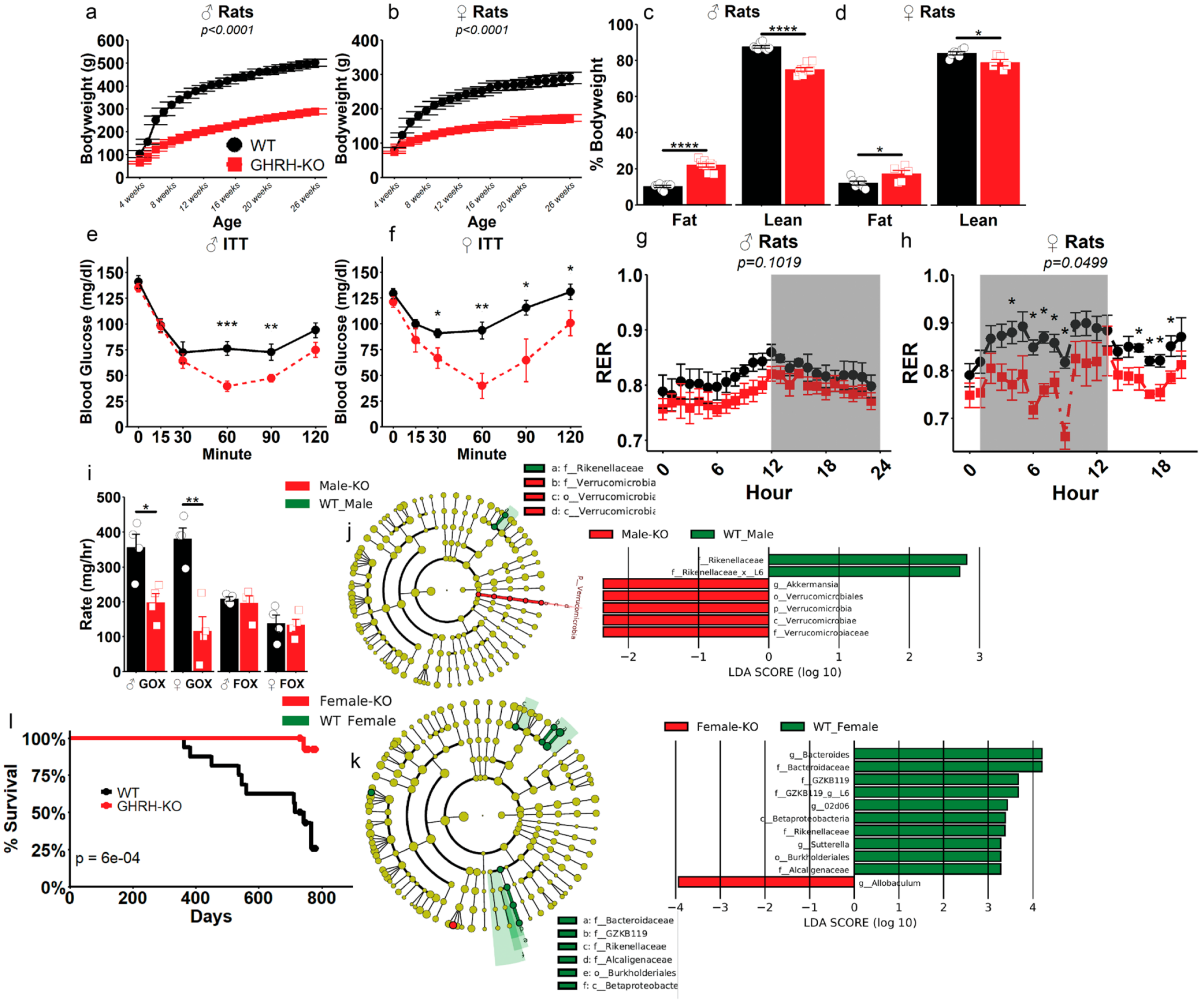
Physiological characteristics and microbiome features of GHRH deficient rats. Weekly bodyweights of male (a) or female (b) rats as indicated. Body composition—percent fat mass and lean mass—in 3‐month‐old male (c) and female (d) rats as indicated. 1 U/kg IPITT in ad‐lib fed male (e) or female (f) rats. Respiratory exchange ratio (RER; VCO_2_/VO_2_) calculated during indirect calorimetry experiments in male (g) and female (h) rats. Rates of glucose (GOX) and fat (FOX) oxidation, calculated as detailed in the methods section, in male and female rats (i). LEfSe results for analysis of differentially abundant taxa within the male (j) or female (k) gut microbiome. Preliminary survival analysis of GHRH‐KO rats (l). Data presented as mean ± SEM with points representing individual rats. *p < 0.05, **p < 0.01, ***p < 0.001 as determined by two‐way repeated measure ANOVA followed by Tukey HSD post hoc test (e, f, g, h) or by two‐tailed *n*‐test with the Welch correction applied (c, d, i). *N* = 4–11 per group. *p*‐values presented represent the main effect of genotype as determined by two‐way repeated measure ANOVA (a, b, g, h) or by log‐rank test (l). *N* = 4–10 (a–k) or *N* = 15–16 per group (7 male WT, 10 male GHRH‐KO, 9 female WT, and 5 female GHRH‐KO; [l]).

GHRH‐KO rats weighed significantly less (Figure 2a,b), displayed significantly greater body fat, and significantly less lean mass as percentages of body weight (Figure 2c,d). Insulin sensitivity was significantly enhanced in GHRH‐KO rats (Figure 2e,f), and while male GHRH‐KO glucose tolerance was improved (Figure S2a) female glucose tolerance was largely unchanged except for 120 min following a glucose challenge (Figure S2b). A tendency for reduced respiratory exchange ratio (RER) was observed in male GHRH‐KO rats, and female RER was significantly lower (Figure 2g,h). Glucose oxidation rate was significantly lower in GHRH‐KO rats while fat oxidation rate was unchanged (Figure 2i). Absolute energy expenditure was significantly lower in GHRH‐KO rats (Figure S2c,d); however, only females displayed reduced energy expenditure after accounting for body weight differences (Figure S2e–h). No differences were seen in alpha or beta diversity metrics of gut microbial abundance (Figure S3a–e); however, qualitative inspection of the composition of the microbial content of our rats revealed some differences in the most abundant phyla within our samples (Figure S3f). LEfSe analysis revealed 7 and 11 differently abundant taxa in GHRH‐KO males and females, respectively (Figure 2j,k). Preliminary assessment of lifespan suggests significantly extended GHRH‐KO rat lifespan, with a median WT lifespan of 728 days and the only GHRH‐KO death being observed at day 742 (Figure 2l and Table S1).

## 3 Discussion

Identification of genetic alterations that extend mammalian lifespan is key to understanding aging and identifying targets to improve human healthspan. In this study, we generated a novel rat model with a targeted deletion in the *Ghrh* gene. These GHRH‐KO rats are GH deficient and exhibit reduced body weight, increased fat percentage, and lower circulating IGF‐I. They also showed heightened insulin sensitivity, improved glucose tolerance in males, and lower reliance on glucose metabolism, as indicated by reduced RERs and glucose oxidation. These metabolic features arose despite only modest differences in gut microbiome composition. Males displayed differential abundance in 7 taxa, and females in 11.

Reduced body size is a hallmark of long‐lived mice with disrupted GH signaling. The ~50% reduction in adult body weight in GHRH‐KO rats is consistent with prior reports in GH‐deficient and dwarf mouse models (Lasher et al. 2024; List et al. 2019). This reduction supports the idea of a growth–longevity tradeoff, where slower development reduces late‐life disease (Bartke et al. 2013). GH treatment in Ames dwarf mice during the first ~7 weeks of life, analogous to human adolescence, normalizes both growth and lifespan (Sun et al. 2017), reinforcing the developmental timing of GH action in aging.

GHRH‐KO rats showed markedly enhanced insulin sensitivity, aligning with data from GH‐deficient (Wiesenborn et al. 2014) and GHRH‐KO mice (Zhang et al. 2020). This trait is common in long‐lived GH‐deficient models (Bartke et al. 2001) and may be crucial for their extended longevity (Masternak et al. 2009). However, insulin sensitivity alone does not guarantee longer lifespan, as some short‐lived models also show it (Nelson et al. 2012). Notably, this enhanced sensitivity occurs despite increased fat mass. Evidence suggests that WAT in these models functions uniquely, with transplants from dwarf mice improving insulin sensitivity in controls (Hill et al. 2016). This implies WAT may play a beneficial metabolic role in GH‐deficient animals, warranting further investigation.

Glucose tolerance findings across GH‐disrupted models vary. Evidence suggests that dwarf mouse glucose tolerance is improved (Darcy et al. 2016; Hill et al. 2016), while past reports in GHRH‐KO mice indicate no change in glucose tolerance (Icyuz et al. 2020; Sun et al. 2013), and mice with deletions of the GH receptor and GH gene display impaired glucose tolerance (Guo et al. 2005; Lasher et al. 2024; List et al. 2019). Here, our GHRH‐KO males were more glucose tolerant while female glucose tolerance was unchanged. Despite mixed glucose tolerance results, reduced glucose usage is consistently observed in GH‐deficient models (Brooks et al. 2007) (Icyuz et al. 2021; Lasher and Sun 2023; Westbrook et al. 2009). Lower RER values and reduced glucose oxidation in our rats reflect this. These findings suggest that reduced glucose reliance, rather than tolerance per se, may be a conserved feature in GH‐related longevity.

While gut microbiome differences between WT and GHRH‐KO rats were limited, they may still influence the phenotype. Males showed greater microbial enrichment, including *Akkermansia*, a genus linked to metabolic health and mucin foraging (Cani et al. 2022) (Davey et al. 2023). In WT males, *Rikenellaceae*—associated with reduced visceral adiposity (Tavella et al. 2021)—was enriched. In females, WT rats showed higher levels of *Bacteroides*, *02d06*, and *Sutterella*, taxa connected to glucose metabolism (Noble et al. 2021; Wang et al. 2020). Female GHRH‐KOs had higher *Allobaculum*, linked to lipid metabolism (Liu et al. 2016; Zheng et al. 2021). These patterns suggest GHRH‐KO rats have microbiomes more geared toward lipid regulation, aligning with their reduced glucose dependency.

Preliminary lifespan data indicate that GHRH‐KO rats may share the extended longevity observed in GH‐deficient mice. Although based on small sample sizes, our findings align with earlier studies in GH‐deficient rats from hypophysectomy or spontaneous mutations (Everitt et al. 1980; Sasaki et al. 2013). Median survival in controls (~728 days) aligns with these prior benchmarks. In contrast, only one GHRH‐KO rat had died by 779 days, comparable with the report of Sasaki et al. (2013) at which point approximately 95% of their long‐lived spontaneous dwarf rat colony remained alive.

Our results differ from studies where lifespan was shortened in rats expressing antisense GH transgenes (Shimokawa et al. 2002) or unchanged in Lewis dwarf rats (Sonntag et al. 2005). The antisense model's reduced survival may be due to off‐target effects, including ectopic transgene expression and high neoplastic mortality, as Shimokawa and colleagues' homozygous rats died almost exclusively from neoplastic disease (2002). In the Lewis model, despite GH deficiency and lower disease burden, lifespan was unaffected—possibly due to unknown aspects of the *dw/dw* mutation, which also alters prolactin levels (Tierney and Robinson 2002). As the specific genetic basis of the Lewis dwarf *dw/dw* mutation remains unknown, it is difficult to attribute differences (or similarities) in their lifespan solely to GH action.

## Author Contributions

Liou Y. Sun and Steven N. Austad conceptualized the study. Liou Y. Sun oversaw overall direction and secured funding. Aron M. Geurts generated mutant animals. Soe Maung Maung Phone Myint designed experiments, collected data, and analyzed data. Kaimao Liu assisted in data collection and animal husbandry. Alexander Tate Lasher assisted with data analysis. Soe Maung Maung Phone Myint and Alexander Tate Lasher drafted the original manuscript. Soe Maung Maung Phone Myint, Alexander Tate Lasher, Steven N. Austad, and Liou Y. Sun edited the manuscript. All authors provided critical feedback that helped shape the research, analysis, and manuscript.

## Conflicts of Interest

The authors declare no conflicts of interest.

## Supporting information


Data S1.

Data S2.

Figure S1.

Figure S2.

Figure S3.

## Data Availability

The data supporting the findings of this study are available from the corresponding author upon reasonable request.
