# Supplementary material for: A Novel GH Deficient Rat Model Reveals Cross‐Species Insights Into Aging
Source: Aging Cell. 2025 Jun 5;24(8):e70126. doi: 10.1111/acel.70126 (PMC12341780; doi:10.1111/acel.70126)
Supplement: Supplementary file 1 — Data S1. [file ACEL-24-e70126-s001.docx]

**Supplemental Table S1** Causes of death observed in WT and GHRH-KO rats

| **Sex** | **Genotype** | **Cause of Death** |
| --- | --- | --- |
| Male | WT | Euthanasia - Low Body Condition |
| Male | WT | Natural Death |
| Male | WT | Natural Death |
| Female | WT | Euthanasia - Ulcerated Tumor |
| Female | WT | Euthanasia - Ulcerated Tumor |
| Female | WT | Euthanasia - Tumor Impairing Mobility |
| Female | WT | Euthanasia - Tumor Impairing Mobility |
| Female | WT | Natural Death |
| Female | WT | Natural Death |
| Female | WT | Natural Death |
| Female | WT | Natural Death |
| Female | GHRH-KO | Euthanasia - Tumor Impairing Mobility |

**Supplemental Table S2** Summary of physiological changes associated with differentially abundant taxa

| **Taxa** | **Enriched Group** | **Physiological Association** | **Reference** |
| --- | --- | --- | --- |
| *Akkermansia* | Male GHRH-KO | ↓ inflammation ↓cholesterol accumulation | Cani et al., 2022; Davey et al., 2023 |
| *Rikenellaceae* | Male WT | ↓ adipose accumulation | Tavella et al., 2021 |
| *Bacteroides* | Female WT | ↑ sugar absorption ↑ glucose metabolism | Noble et al., 2021; Wang et al., 2020 |
| *02d06* |  |  |  |
| *Sutterella* |  |  |  |
| *Allobaculum* | Female GHRH-KO | ↑lipid metabolism | Liu et al., 2016; Zheng et al., 2021 |
