# Supplementary material for: A Novel GH Deficient Rat Model Reveals Cross‐Species Insights Into Aging
Source: Aging Cell. 2025 Jun 5;24(8):e70126. doi: 10.1111/acel.70126 (PMC12341780; doi:10.1111/acel.70126)
Supplement: Supplementary file 2 — Data S2. [file ACEL-24-e70126-s005.docx]

**Supplementary Methods**

*Development of Mutant Rats*

The CRISPR-Cas system was employed to generate mutations in the rat gene coding for GHRH. Five target sites with high efficiency scores in exon 3 of the rat *Ghrh* gene were identified using the CRISPR design feature of Benchling (<https://www.benchling.com/>). Synthetic modified single guide RNA (sgRNA) for these sequences were acquired from Synthego and their efficacy was validated as previously detailed (Challa et al., 2021). Validated sgRNA-Cas9 ribonucleoprotein complexes were electroporated into Sprague-Dawley rat embryos and then transferred to pseudopregnant female rats to obtain founder animals harboring mutations in the *Ghrh* gene. The founder animals were screened by PCR and heteroduplex mobility assay as described (Challa et al., 2016).

*Animal Husbandry*

Mutant rats and wild-type littermates were maintained on the outbred Sprague-Dawley genetic background. Tail biopsies were collected from 18 day old rats, DNA was extracted using a previously described procedure (Truett et al., 2000), and genotyping was performed by PCR using the forward primer 5’- GGCTGAGGTGACAGGGAAAT-3’ and reverse primer 5’- GACAACAAAGGGGGACCTTA-3’. PCR products were resolved by polyacrylamide gel electrophoresis. Rats were housed by sex and genotype at a density according to their body weight with no more than 2 per cage when they reach a weight of more than 400 grams. Animals were maintained in a pathogen-free facility on a standard 12-12 light and dark cycles at 20-23 degrees C. All animals had *ad-libitum* access to standard rodent chow (NIH-31 Rat and Mouse diet, 18% protein, 4% fat) and drinking water except when the rats have been fasted for specific experiments as indicated. Both sexes of rats have been utilized for this study and all experimental protocols were approved by the University of Alabama at Birmingham Institutional Animal Care and Use Committee (IACUC).

*RNA extraction and RT-qPCR*

3–4-month-old male and female rats with *ad-lib* access to rodent chow were euthanized by CO_2_-induced deep anesthesia followed by decapitation. Pituitaries were dissected and were rapidly frozen on dry ice. RNA was extracted from frozen pituitaries using the TRIzol reagent (Invitrogen catalog# 15596026) following the manufacturer’s protocol. The resulting RNA pellet was re-extracted using the RNeasy plus mini kit (Qiagen) to yield high quality, DNA-free RNA. 100ng total RNA was reverse transcribed in a 20µL reaction containing 50U M-MuLV reverse transcriptase (New England Biolabs catalog# M0253), 1x reverse transcription buffer, 6µM random hexamers, 0.5µM dNTPs, and 8U of murine RNase inhibitor (New England Biolabs catalog# M0314). Reverse transcription reactions were incubated for 5 minutes at 25 degrees C, 60 minutes at 42 degrees C, and 20 minutes at 65 degrees C. qPCR reactions were carried out using the product of these reactions as template with Luna qPCR master mix (New England Biolabs catalog# M3003) on a Quantstudio 3. The following primers were used: *Gh1* F 5’- TTCGAGCGTGCCTACATTCC-3’; *Gh1* R 5’- GCGAAGCAATTCCATGTCAGTT-3’; *Actb* F 5’-ACCCGCCACCAGTTCGCCAT-3’; *Actb* R 5’- CGGCCCACGATGGAGGGGAA-3’. Fold change was calculated using the using the 2^−ddCt^ calculation with *Actb* as an endogenous control.

*Body Composition Analysis*

Body composition was analyzed in rats at 3 months of age using the EchoMRI Whole Body Composition Analyzer for rats in conjunction with EchoMRI 2018 Body Composition Analyzer software. Unanesthetized rats were placed into the Body Composition Analyzer holding tube in a prostrate position and scanned. Data obtained included lean mass and fat mass. Body composition measurements were conducted by the University of Alabama at Birmingham Small Animal Phenotyping Core.

*Insulin & Glucose Tolerance Tests*

Four-to-five-month-old rats were assessing glucose homeostasis. For insulin tolerance tests (ITT) rats with *ad-libitum* access to standard rodent diet were administered 1 IU/kg insulin (Humulin-R, Eli-Lily) by intraperitoneal (IP) injection. Food was withdrawn immediately prior to ITT to prevent eating during the test. For glucose tolerance tests (GTT), rats were fasted overnight for 16 hours and were administered 1 g/kg of glucose by IP injection. Blood glucose measurements from a tail nick were measured using a handheld blood glucometer (AgaMatrix PRESTO) immediately prior to injection (at “minute 0”) and after injection at the indicated time points.

*Serum IGF-I Content Analysis*

Whole blood was collected from 1-year-old non-fasted rats via the tail vein after inducing anesthesia with isoflurane (3%) and allowed to clot for 15 minutes at room temperature. Serum was collected by centrifugation (3000xg at 4^o^C for 10 minutes). The collected serum was stored at -80^o^C until analysis. Serum IGF-I content was determined using a Rat IGF-I ELISA Kit (Crystal Chem, catalog# 80573) following the manufacturer’s protocol.

*Indirect calorimetry*

Two-year-old rats were individually maintained for 1 week prior to all indirect calorimetry experiments to acclimate to the stress of single housing. Following this acclimation period, Rats were individually housed in PhenoMaster indirect calorimetry chambers (TSE Systems) for approximately 3 days, with flow rates of 2.0 (females) or 2.5 (males) liters per minute. Data from the first 48 hours were omitted from analysis, as this period was considered an additional acclimation period to allow adjustment to a new housing environment. The male data collection period began at 6am, the first hour of the animal facility’s light cycle, and the female data collection period began at 5pm, the final hour of the animal facility’s light cycle. Oxygen consumption (VO2) and carbon dioxide production (VCO2) data were recorded every 27 minutes for each animal. Respiratory exchange ratios (RER), glucose oxidation (GOx), fat oxidation (FOx), and energy expenditure were calculated from VO2 and VCO2 values as previously detailed (Lasher & Sun, 2023; Nagarajan, Lasher, Morrow, & Sun, 2024).

*Fecal sample collection and 16s rRNA Sequencing*

Nine-ten-month-old GHRH-KO rats and wild-type controls were singly placed into autoclaved cages and allowed to pass fecal matter. 2-3 fecal pellets were then collected per rat into 1.5 ml tubes and stored in ice. The samples were then placed into -80^o^C freezer for storage before sending them out to UAB Microbiome Resource for 16s rRNA sequencing. DNA was extracted using a Fecal DNA Isolation Kit (Zymo Research) and used for PCR to amplify the V4 region of the 16S rRNA gene. The PCR products were then run through an agarose gel, after which the bands were visualized under UV. The bands were then excised and purified using a QIAquick gel extraction kit. The product is then sequenced via Illumina MiSeq platform.

*Bioinformatic Analysis of microbiomes*

The raw data files (fastq) files were acquired from the UAB Microbiome Resource following the sequencing process. Quantitative insights for microbial ecology 2 (QIIME2) environment was used to both import the fastq data files and perform analysis of the alpha and beta diversity of the samples. DADA2 was used as the denoising method to make sure that the reads were filtered and replicates and chimera removed. QIIME2 phylogeny plugin was used to construct a phylogenetic tree and aligned with our sample data using align-to-tree-mafft-fasttree. Rarefaction was also performed to ensure that the same sequence depth was used for all samples. Taxonomic classification was conducted using the Greengenes database, a naïve Bayes classifier. The alpha diversity metrics such as Faith’s phylogenetic diversity and Observed ASV’s were analyzed.

Assessment of beta diversity was performed via Unweighted Unifrac distance method after which Principal Coordinate Analysis (PCoA) plots were made using QIIME2 and phyloseq in R. Permutational multivariate analysis of variance (PERMANOVA) was used to determine the statistical differences between the separate groups. Linear discriminant analysis by effect size (LEfSe) was used to analyze the differentially present taxa amongst the groups, using the Dokdo Python package (<https://github.com/sbslee/dokdo>).

*Survival analysis*

Following the experiments described above, the animals were maintained under standard husbandry conditions and were monitored daily by laboratory personnel and twice per week by the animal resource personnel at the University of Alabama at Birmingham for health, who are unaffiliated with our lab and are blinded to our experimental design. Animals were marked dead on the date they were found dead, or on the date of euthanasia if animal resource personnel determined the animal had a condition which would likely lead to death before the next health inspection. This later scenario occurred in one male WT rat where veterinary staff determined its low body condition would likely lead to before the next inspection, four female WT rats where veterinary staff observed ulcerated tumors (two rats) and tumors that impaired the animal’s mobility (two rats), and in one GHRH-KO rat where veterinary staff observed a tumor that impaired mobility. These observations are summarized in Supplementary Table S1. Animals still alive at the preparation of this manuscript were treated as right censored.

*Statistical Analysis*

Two group means were compared using the unpaired two-tailed t-test with the welch correction applied. When comparing the means of several groups, factorial repeated measure ANOVA and PERMANOVA was used. Rat survival was visualized by Kaplan-Meier survival curves and group survival was compared using the log-rank test. P <0.05 was considered significant for all means and all analysis were performed and figures generated via R. Lefse Analysis was performed using Dokdo Python package (<https://github.com/sbslee/dokdo>) and the cladogram generated via the same package as well.

**References**

Challa, A. K., Boitet, E. R., Turner, A. N., Johnson, L. W., Kennedy, D., Downs, E. R., . . . Kesterson, R. A. (2016). Novel Hypomorphic Alleles of the Mouse Tyrosinase Gene Induced by CRISPR-Cas9 Nucleases Cause Non-Albino Pigmentation Phenotypes. *PLoS One, 11*(5), e0155812. doi:10.1371/journal.pone.0155812

Challa, A. K., Stanford, D., Allen, A., Rasmussen, L., Amanor, F. K., & Raju, S. V. (2021). Validation of gene editing efficiency with CRISPR-Cas9 system directly in rat zygotes using electroporation mediated delivery and embryo culture. *MethodsX, 8*, 101419. doi:10.1016/j.mex.2021.101419

Lasher, A. T., & Sun, L. Y. (2023). Distinct physiological characteristics and altered glucagon signaling in GHRH knockout mice: Implications for longevity. *Aging Cell*, e13985. doi:10.1111/acel.13985

Nagarajan, A., Lasher, A. T., Morrow, C. D., & Sun, L. Y. (2024). Long term methionine restriction: Influence on gut microbiome and metabolic characteristics. *Aging Cell, 23*(3), e14051. doi:10.1111/acel.14051

Truett, G. E., Heeger, P., Mynatt, R. L., Truett, A. A., Walker, J. A., & Warman, M. L. (2000). Preparation of PCR-quality mouse genomic DNA with hot sodium hydroxide and tris (HotSHOT). *Biotechniques, 29*(1), 52, 54. doi:10.2144/00291bm09
