# Supplementary figures and images for: A Novel GH Deficient Rat Model Reveals Cross‐Species Insights Into Aging
Source: Aging Cell. 2025 Jun 5;24(8):e70126. doi: 10.1111/acel.70126 (PMC12341780; doi:10.1111/acel.70126)

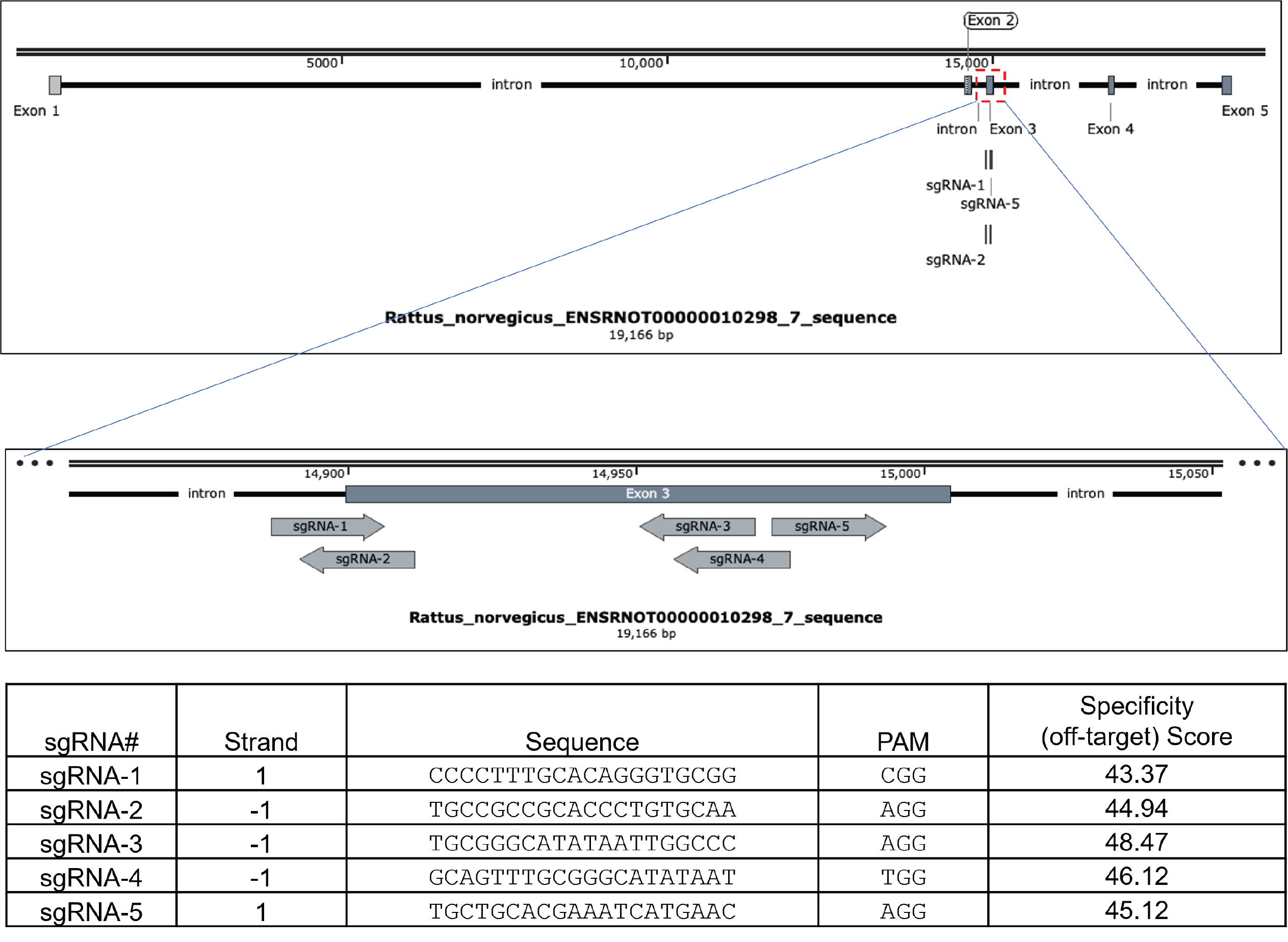

Supplement: Supplementary file 3 — Figure S1. [file ACEL-24-e70126-s004.png]

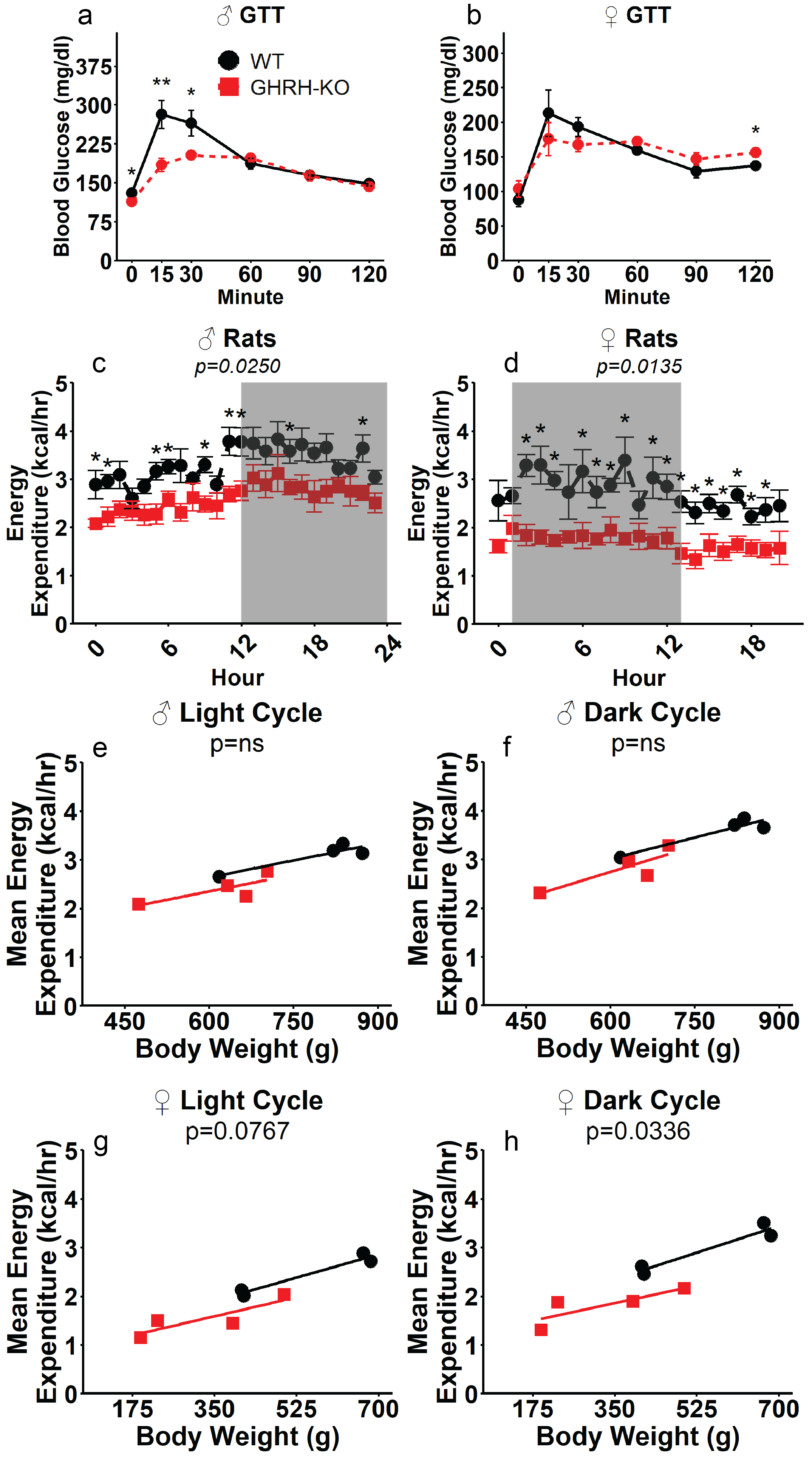

Supplement: Supplementary file 4 — Figure S2. [file ACEL-24-e70126-s003.png]

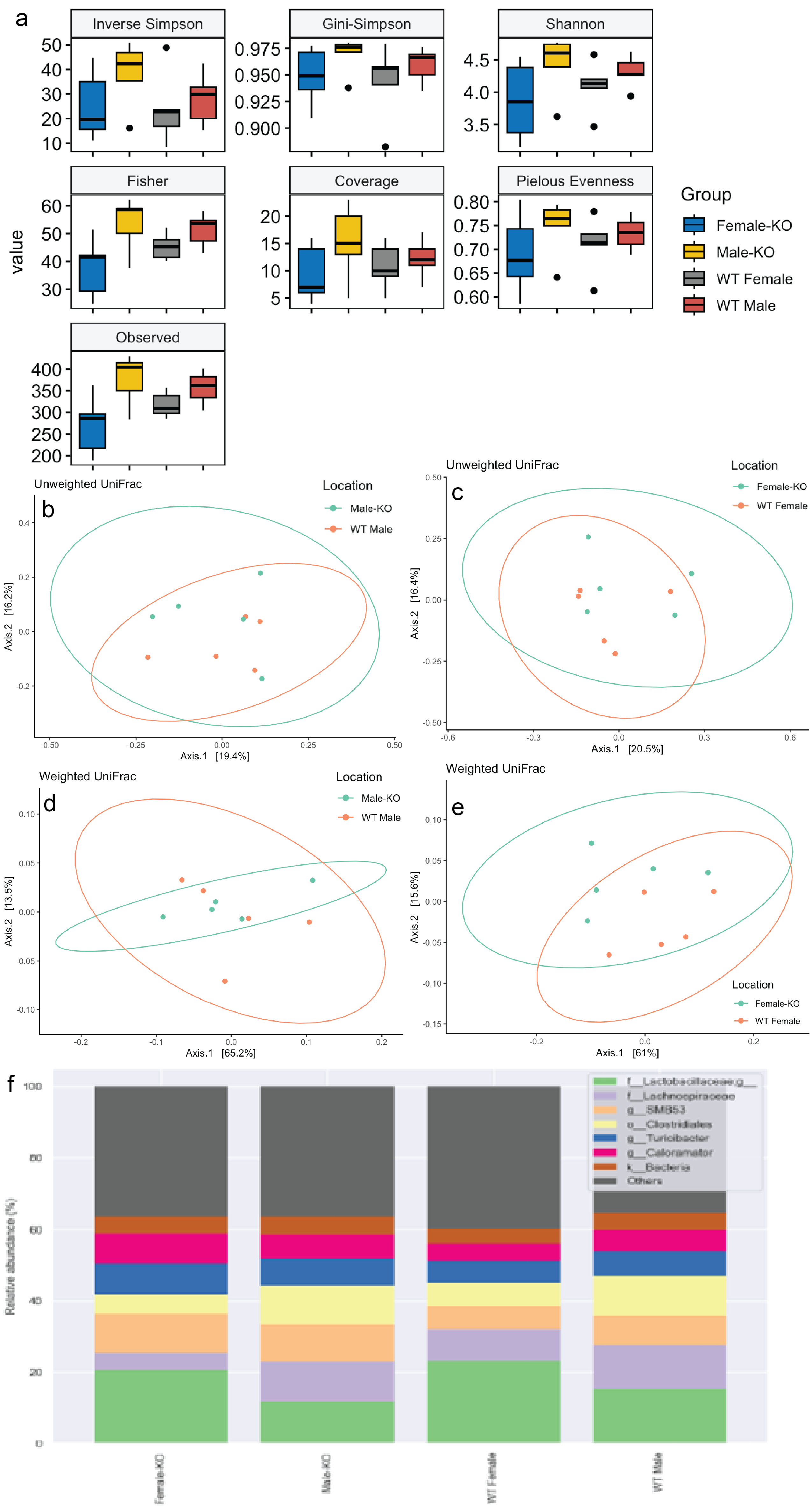

Supplement: Supplementary file 5 — Figure S3. [file ACEL-24-e70126-s002.png]
